# Airway leech infestation masquerading as a tumor: a rare case report

**DOI:** 10.3389/fonc.2025.1572523

**Published:** 2025-06-16

**Authors:** Pengyu Li, Xianmeng Chen, Wanling Chen, Yunchao Huang, Xiangxiu Tan, Ying Chen

**Affiliations:** ^1^ Thoracic Department I, The Third Affiliated Hospital of Kunming Medical University, Peking University Cancer Hospital Yunnan (Yunnan Cancer Hospital), Kunming, China; ^2^ Northeast Yunnan Central Hospital of Kunming Medical University Thoracic Department, Northeast Yunnan Central Hospital, Zhaotong, China

**Keywords:** leech infestation, respiratory tumor, airway, thoracic, case report

## Abstract

Airway obstruction is a ventilatory dysfunction caused by internal and external diseases of the respiratory tract, which is usually considered a life-threatening clinical emergency. Traditionally, airway obstruction can be caused by respiratory tumors, trauma, and foreign bodies, among which tumors are the most common. Consequently, physicians may prioritize tumor-related diagnoses, potentially leading to the misdiagnosis of rare non-neoplastic conditions. Here, we report a case of a 61-year-old adult traveler who initially presented with mild dyspnea and a bronchial space-occupying lesion on a CT scan, raising suspicion of an airway tumor. However, further examination confirmed the diagnosis of a living leech infestation in the bronchus, resulting from a trip to Laos. This case highlights the importance of thorough history-taking and critical thinking in clinical diagnosis, urging physicians to avoid overreliance on intuition or experience alone.

## Introduction

Conventionally, respiratory tumors and foreign bodies have been considered the major causes of airway obstruction, though other rare conditions may exist (1, 2). Airway obstruction is defined as the blockage of any portion of the airway, a condition that can become a life-threatening emergency under certain circumstances. However, physicians, particularly those in oncology specialty hospitals, often prioritize tumor-related diagnoses when encountering bronchial space-occupying lesions on CT scans. This approach may potentially lead to the oversight of rare conditions such as bronchial leech infestation, as illustrated in this rare case report.

## Case study

**Figure 1:**
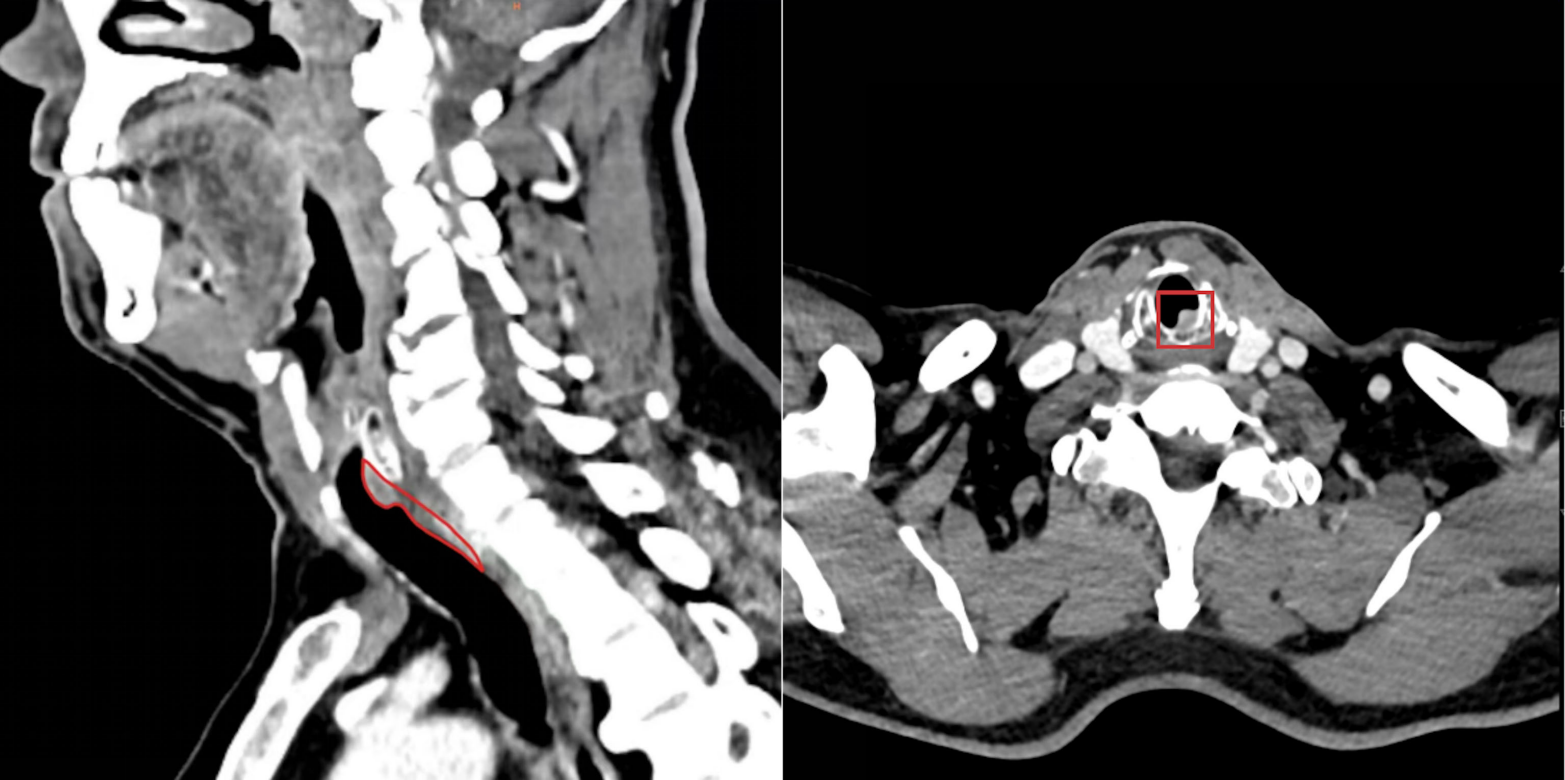
A nodule of undetermined origin was present in the posterior wall of the subglottic region of the patient’s larynx.

The patient was a 61-year-old man from Pu’er City, Yunnan Province, China. In October 2024, he presented to the outpatient department of Thoracic Surgery at Yunnan Cancer Hospital with mild dyspnea but no cough or hemoptysis. A CT scan revealed an obscure foreign body (8 cm in length and 1.5cm in diameter) in the posterior wall of the subglottic bronchus (**Figure 1**), initially suspected to be an endotracheal tumor.

**Figure 2:**
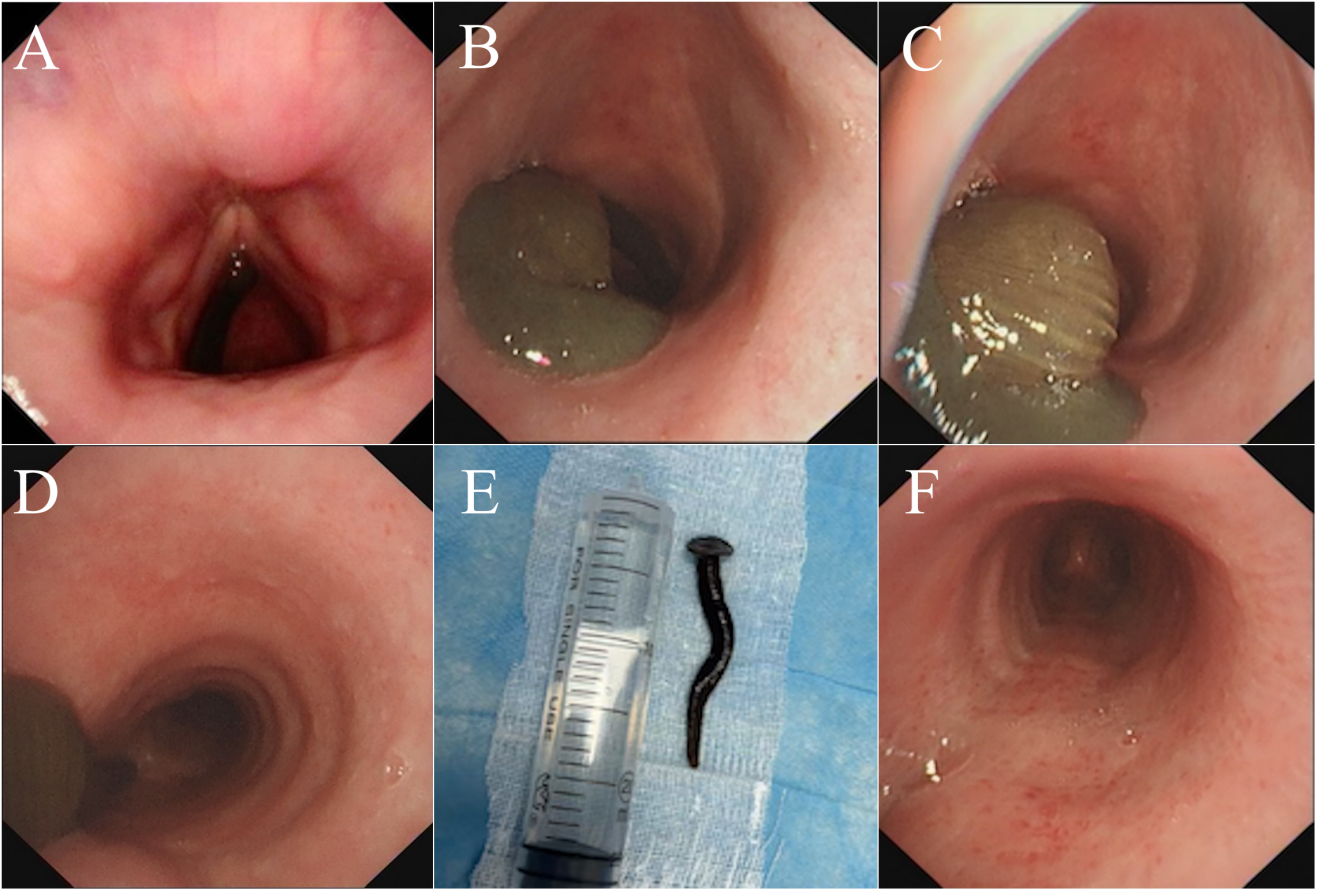
Results of electronic fiber laryngoscopy and bronchoscopy of the patient. **(A)** No tumors or foreign bodies were found in the throat. **(B)** As the examination site was deepened, a living brown verminous foreign body was found on the posterior wall of the subglottic bronchus. **(C)** The entire foreign body was located within the bronchus. Its tail, positioned under the glottis, was shaped like a round sucker. **(D)** The top of the foreign body was located above the bifurcation of the bronchus. **(E)** The foreign body was identified as a live leech of the species *Dinobdella ferox* (Blanchard, 1896) (measuring 8 cm × 1.5 cm), which had a flat, spindle-shaped body with a dark and green dorsum often displaying irregular markings. The surface was smooth with segmented rings. Based on its body length, the leech was estimated to be approximately 2 months old. **(F)** After the leech was removed, the surface of the attachment site exhibited a rough and irregular texture.

For further evaluation, the physicians planned a biopsy of the intrabronchial space-occupying lesion after a routine blood analysis test, and the results showed markedly elevated fibrinogen degradation product (FDP) and D-dimer levels. Fiberoptic laryngoscopy excluded thyroid adenomas and pharyngeal foreign bodies (**Figure 2A**). Subsequent fiberoptic bronchoscopy of the subglottic bronchus revealed a soft, brown, mobile, and malleable verminous foreign body (**Figure 2B**), ruling out the initial neoplastic diagnosis. The entire living foreign body was completely inside the bronchus. Its tail side, located under the glottis, resembled a round sucker (**Figure 2C**), attached to the bronchial mucosa, while the top was positioned above the bifurcation of the bronchus (**Figure 2D**). The physicians paralyzed it with *lidocaine* (a topical anesthetic) immediately and successfully removed it using a snare under an electronic bronchofiberscope while the patient was under general anesthesia. Based on its morphological features, we identified it as a leech, measuring 8 cm in length and 1.5 cm in diameter (**Figure 2E**). The endotracheal surface, the attachment site, exhibited a rough and irregular texture with transparent secretions. The surrounding mucosal tissue displayed mild edema and was accompanied by scattered petechial hemorrhages (**Figure 2F**).

For further confirmation, we reviewed the patient’s medical history and found that he had traveled to Laos 3 months ago and had drunk water from local streams several times. More importantly, he had experienced severe choking during his drinking. Ultimately, by consulting a parasitologist, we confirmed it to be a leech of the species *Dinobdella ferox* (Blanchard, 1896).

It had the same morphological features. Its color was dark and green, completely without markings. The head was small, and the tail sucker was very large. Its eyes were small, deeply arranged, and indistinct. Segment VIII was divided into four rings, often enlarged and typically exhibiting a crescent-shaped groove on the ventral surface. There were 16 complete segments (segments IX–XXIV), each comprising five rings, although the terminal segments may occasionally display four rings.

After the leech was removed, the patient’s symptoms disappeared, and he was discharged on the same day. After 1 month of follow-up, the patient was in good health.

## Discussion

Both respiratory tumors and foreign bodies can cause airway obstruction. However, physicians, particularly those in oncology specialty hospitals, often prioritize tumor-related diagnoses, potentially overlooking rare conditions such as bronchial leech infestation. Although imaging findings may resemble those of tumors, the biological characteristics of leeches can lead to distinct clinical manifestations, which may aid in reaching the correct diagnosis.

Leeches are blood-feeding hermaphrodite parasites belonging to the class Clitellata of the phylum Annelida. They inhabit diverse environments, including freshwater, estuaries, rivers, ponds, lakes, and seas (3). The blood-sucking leeches are common in the damp forests of the subtropical and tropical Indo-Pacific region, and members of the blood-sucking leeches are abundant in Laos and adjacent countries of Southeast Asia (4).

Leeches are difficult to detect in their larval stage, making it easy for them to enter the human body through the oral or nasal route during activities such as bathing or drinking. Leeches often parasitize the esophagus and pharynx. However, if one leech is stuck between the entrance of the esophagus and the respiratory tract, it can enter the lower respiratory tract when the person coughs or breathes. As aerobic mollusks, the bronchial mucosa provides an ideal environment for the survival of larval leeches, offering sufficient oxygen, rich blood flow, suitable temperature, and appropriate humidity. Based on the patient’s medical history, his exposure to untreated stream water in Laos supports the hypothesis of accidental ingestion. The leech then attached to the bronchial mucosal surface and, within 2–3 months, grew in size within his respiratory tract, eventually causing respiratory obstruction.

As an anesthetic secreted by leeches (5), hirudin can reduce bronchial irritation and alleviate the patient’s sensation of a foreign body. As a result, patients may remain asymptomatic during the early stages of infestation. Additionally, hirudin causes excessive bleeding at the attachment site, enabling the leech to ingest up to nine times its own body weight in blood, which can lead to severe anemia (6). One case report described a 15-year-old girl who developed anemia due to leech infection (7). In this case, the patient’s blood test results showed significantly elevated levels of FDP and D-dimer, which may be attributed to the anticoagulant secreted by the leech (**Table 1**).

**Table 1:** The CBC and coagulation findings of the patient with reference intervals.

No.	Test item	Result		Unit	Reference range
1	*White blood cells (WBC)	3.65		×10^9^/L	3.5–9.5
2	Lymphocyte ratio	39.7		%	20–50
3	Monocyte ratio	7.9		%	3–10
4	Neutrophil ratio	46.6		%	40–75
5	Eosinophil ratio	4.4		%	0.4–8
6	Basophil ratio	1.4	↑	%	0–1
7	Lymphocyte absolute count	1.45		×10^9^/L	1.10–3.20
8	Monocyte absolute count	0.29		×10^9^/L	0.10–0.60
9	Neutrophil absolute count	1.7	↓	×10^9^/L	1.80–6.30
10	Eosinophil absolute count	0.16		×10^9^/L	0.02–0.52
11	Basophil absolute count	0.05		×10^9^/L	0–0.06
12	Fibrinogen degradation product (FDP)	5.08	↑	μg/L	<5
13	D-dimer	1.38	↑	mg/L	0–0.55

The symbol (↑) means the actual value of the patient's test result is exceeds the reference range. The symbol (↓) means the actual value of the patient's test result is below the reference range.

In summary, with the improvement of living standards and hygiene awareness among residents, cases of leech infection are gradually declining or becoming rare in local communities. However, the frequency of such infections among travelers is increasing. Therefore, travelers visiting leech habitats should pay greater attention to water hygiene and safety.

In addition, bronchial leech infestation, being a rare condition, can be asymptomatic in the early stages, making it difficult to detect through auxiliary examinations or imaging equipment. In later stages, when the leech reaches a certain size, it may also be mistaken for a tumor if diagnosed solely based on imaging data. Therefore, for patients with bronchial space-occupying lesions, bronchoscopic pathological biopsy offers significant advantages in achieving a definitive diagnosis, facilitating differential diagnosis, and guiding treatment.

## Conclusion

This case underscores the importance of travelers being informed about infectious disease risks at their destination. Although well-established treatments are available, prevention remains paramount. Physicians should thoroughly review epidemiological histories and relevant hygiene details, and appropriate diagnostic tests are essential to avoid misdiagnosis or missed diagnoses. Most importantly, clinicians must base their judgments on factual evidence rather than relying predominantly on subjective experience or assumptions.

## Case characteristics

1. An adult male drank untreated water multiple times during his travel in Laos within a 3-month period, eventually developing an endotracheal leech infection, which is an extremely rare condition among travelers.
2. Initially, a tracheal tumor was suspected based on imaging findings, but bronchoscopy ultimately confirmed the presence of a live leech causing endobronchial infection.
3. The larval leech initially remained asymptomatic during its growth phase until its increasing size led to clinical manifestations.

## Data Availability

The original contributions presented in the study are included in the article/Supplementary Material. Further inquiries can be directed to the corresponding authors.
